# Random forests for the analysis of matched case–control studies

**DOI:** 10.1186/s12859-024-05877-5

**Published:** 2024-08-01

**Authors:** Gunther Schauberger, Stefanie J. Klug, Moritz Berger

**Affiliations:** 1 Chair of Epidemiology, TUM School of Medicine and Health, Technical University of Munich, Munich, Germany; 2 Institute of Medical Biometry, Informatics and Epidemiology, Faculty of Medicine, University of Bonn, Bonn, Germany

**Keywords:** Conditional logistic regression, Conditional logistic regression forests, Matched case–control studies, Machine learning, Random forest, CLogitForest

## Abstract

**Background:**

Conditional logistic regression trees have been proposed as a flexible alternative to the standard method of conditional logistic regression for the analysis of matched case–control studies. While they allow to avoid the strict assumption of linearity and automatically incorporate interactions, conditional logistic regression trees may suffer from a relatively high variability. Further machine learning methods for the analysis of matched case–control studies are missing because conventional machine learning methods cannot handle the matched structure of the data.

**Results:**

A random forest method for the analysis of matched case–control studies based on conditional logistic regression trees is proposed, which overcomes the issue of high variability. It provides an accurate estimation of exposure effects while being more flexible in the functional form of covariate effects. The efficacy of the method is illustrated in a simulation study and within an application to real-world data from a matched case–control study on the effect of regular participation in cervical cancer screening on the development of cervical cancer.

**Conclusions:**

The proposed random forest method is a promising add-on to the toolbox for the analysis of matched case–control studies and addresses the need for machine-learning methods in this field. It provides a more flexible approach compared to the standard method of conditional logistic regression, but also compared to conditional logistic regression trees. It allows for non-linearity and the automatic inclusion of interaction effects and is suitable both for exploratory and explanatory analyses.

## Background

A random forest is a machine learning technique first proposed by ﻿Breiman [1]. It is an ensemble learning algorithm, where several individual models (or base-learners) are combined into a final estimator. The individual learners in random forests are trees, for example, classification and regression trees (CART) [2]. CARTs are a partitioning technique which divides the covariate space using binary splits. For each partition, a different prediction is calculated, for example by averaging over all outcome values or computing the relative frequencies of the single outcome categories among the observations in the respective partition. Their tree structure can be visualized via dendrograms, where each split in a particular variable generates a new branch.

Random forests use the machine learning principle of ’bagging’ [3] (i.e. bootstrap aggregating). In bagging, bootstrap samples [4] are drawn from the original training data. Each bootstrap sample is then used to estimate an individual model, a so-called base-learner. The final model is built by an aggregation of all individual base-learners. The main idea of ensemble learners in general and of bagging in particular is that the variability of the ensemble will be smaller than the variability of the single base-learners. Accordingly, the variability of random forests will be smaller than the variability of trees. While the commonly high variability of trees can be a disadvantage if trees are used individually, it serves as a strength if trees are used as base-learners in random forests. The more variable the individual learners in ensemble methods are, the lower the expected loss of the ensemble they constitute (assuming a constant individual loss). Furthermore, ensemble learners will be better if the individual base-learners are less correlated with each other. Therefore, bagging methods seek to de-correlate the individual base-learners to improve the ensemble model by using bootstrap samples instead of the original data. Random forests use a second step of randomization to de-correlate the individual base-learners. When a new partition is searched for in an individual tree, only a random subset of all variables is used. This automatically leads to somewhat different (i.e., less correlated) trees, as specific structures in one tree become impossible to find in other trees.

Matched case–control studies are an important tool for the generation of real-world evidence (RWE) through real-world data (RWD) [5]. They are particularly helpful for relatively rare diseases, as they allow researchers to achieve a comparatively large number of diseased persons in the data. For a given number of cases, controls are searched for which are not suffering from the disease of interest, but are similar or equal to the corresponding cases in other variables (i.e. the matching variables). Popular matching variables are sex, age and area of residence. Cases and their corresponding controls then constitute strata which cannot be treated as independent observations in a logistic regression model (with the respective disease as the outcome). A downside of matching is that matching on potential confounders can lead to a selection bias, which causes cases and controls to be more similar in the respective data set than in the underlying population. Recently, Mansournia and Poole ﻿[6] highlighted that the effects of this selection bias are prone to misinterpretations and not fully understood yet. However, while typical confounders like sex or age potentially could also be adjusted for within the data analysis, this is not feasible for matching variables with many categories like area of residence [7, 8]. Therefore, methods which account for matching are needed in many applications.

The standard way of analyzing matched case–control studies while accounting for matching is conditional logistic regression. It can integrate covariates for confounder adjustment and uses strata-specific intercepts in order to account for possible strata effects. Estimation is done using a conditional likelihood approach, which eliminates the strata-specific parameters from the objective function. Building upon this principle, Schauberger et al. [9] proposed conditional logistic regression trees as an alternative to ordinary conditional logistic regression. Conditional logistic regression trees are embedded into the general framework of conditional logistic regression and, therefore, automatically include adjustment for the matched strata. Compared to the standard method of conditional logistic regression, conditional logistic regression trees do not require the strict assumption of a linear functional association between covariates and the outcome and allow for an automatic and data-driven way of including interactions between the single variables.

Conditional logistic regression trees are the first machine learning technique for matched case–control studies that goes beyond a simple linear and additive modeling of the single covariates. However, alternatives to ordinary conditional logistic regression with respect to the estimation concept have been proposed.Avalos et al. [10] and Reid and Tibshirani [11] proposed to use $L_1$ penalization techniques which allow for an automatic variable selection during the estimation process. Zetterqvist et al. [12] proposed the concept of doubly-robust conditional logistic regression. Shomal Zadeh et al. [13] proposed matched forest, which is also a random forest method for matched case–control studies. Matched forest is a method for variable selection, particularly focusing on high-dimensional matched case–control studies. However, matched forests are not designed to actually perform modelling of the data, the actual analysis of the selected variables is subsequently done via conditional logistic regression. Furthermore, matched forests are limited to matched pairs while the method proposed in this manuscript addresses the more general matching of one case to several controls. As outlined below, the method proposed in this manuscript will allow for a discrimination between a dedicated exposure variable which is of main interest and other (confounding) variables which are included in the analysis. In matched forests, such a discrimination is not considered.

In general, the main goal behind the analysis of matched case–control studies can be either exploratory or explanatory. While in an explanatory analysis, the researcher is mostly interested in the effect of a dedicated exposure variable, an exploratory analysis is designed to create hypotheses about which variables are potential risk (or protective) factors for the disease at hand and which variables are negligible.

In this manuscript, we propose a random forest method that builds upon the conditional logistic regression trees proposed in Schauberger et al. [9]. Analogous to conditional logistic regression trees, the proposed method has an option to specify a dedicated exposure variable, but can also be used for a purely exploratory analysis. Compared to conditional logistic regression trees, it provides a more stable estimation and increased flexibility in the potential functional forms of the covariates. Beside the basic algorithm, it is described how a separate exposure effect together with confidence intervals can be estimated within the random forest framework. Furthermore, an accompanying method of variable importance is introduced and an implementation of the proposed method in R is presented. The proposed method is compared to ordinary conditional logistic regression and conditional logistic regression trees in a simulation study using different types of data-generating processes. For further illustration, the method is applied to data from the TeQaZ study, a matched case–control study on cervical cancer.

## Methods

### Conditional logistic regression

Conditional logistic regression [14] is applied if, for example in a matched case–control study, the observations come in *n* strata of size $m_i, i=1,\ldots ,n$. The case–control status defines the binary outcome $y_{ij} \in \{0,1\}$ where $y_{ij}=1$ for cases and $y_{ij}=0$ for controls. By design, a stratum contains one case and one or more controls which are matched to this case. We assume the number of cases per stratum to be restricted to one, i.e. $\sum _{j=1}^{m_i} y_{ij} = 1$. For these data, an ordinary model of conditional logistic regression (CLR) can be denoted as

1 $$\begin{aligned} log\left( \dfrac{P(y_{ij}=1)}{P(y_{ij}=0)}\right) = \alpha _{i} + \textbf{z}_{ij}^T\varvec{\gamma }, \quad i = 1,\ldots ,n, ~ j = 1,\ldots ,m_i\,. \end{aligned}$$

The strata-specific intercepts $\alpha _i$ represent strata effects, which describe the similarity between all observations in one stratum with respect to the matching criteria. All other variables are modeled using the simple linear term $\textbf{z}_{ij}^T\varvec{\gamma }$ with coefficient vector $\varvec{\gamma }$ and covariate vector $\textbf{z}_{ij}$.

In cases of explanatory models, where we are interested in one particular of the variables (i.e. the exposure variable), we separate this variable in our mathematical notation. For the rest of the manuscript, the exposure variable will be denoted as *x* (with treatment effect $\beta$) while all further covariates are collected in the vector $\textbf{z}$. Using this notation, CLR is denoted as

2 $$\begin{aligned} \log \left( \dfrac{\text {P}(y_{ij}=1)}{\text {P}(y_{ij}=0)}\right) = \alpha _{i} + x_{ij}\beta + \textbf{z}_{ij}^T\varvec{\gamma }, \quad i = 1,\ldots ,n, ~ j = 1,\ldots ,m_i\,. \end{aligned}$$

For the estimation of such a model, the corresponding conditional likelihood is used where the stratum-specific intercepts $\alpha _{i}$ are eliminated from the likelihood by conditioning on the number of cases per stratum. For further details see Schauberger et al. [9] or Breslow and Day [15].

### Conditional logistic regression trees

The method of conditional logistic regression trees [9] (CLogitTree) was introduced as an alternative to CLR. It has the advantage, that the assumptions for the functional relationship between the covariates (i.e. the variables contained in $\textbf{z}$) and the outcome are much weaker. In particular, no linear relationship is assumed, and interactions are included automatically in a data-driven manner.

Conditional logistic regression trees take advantage of the fitting process of CLR models via the conditional (log-)likelihood. They start with an initial model that only contains the strata-specific intercepts and a separate exposure effect and then gradually evolve by finding optimal partitions of the covariate space. The final model can be denoted as

3 $$\begin{aligned} \quad \log \left( \dfrac{\text {P}(y_{ij}=1)}{\text {P}(y_{ij}=0)}\right) = \alpha _{i} + x_{ij}\beta + f(\textbf{z}_{ij}), \end{aligned}$$

where $f(\textbf{z}_{ij})$ represents the effect of the variables collected in $\textbf{z}$ and can be displayed as a tree via dendrograms. In an explorative setting, where we are not interested in a particular exposure variable, the separate term $x_{ij}\beta$ containing a linear exposure effect can be omitted. The tree is embedded into the CLR framework via (products of) indicator functions, which represent the terminal nodes of the tree $S_1,\ldots , S_t$. Accordingly, the tree $f(\textbf{z}_{ij})$ can in general be denoted as

4 $$\begin{aligned} f(\textbf{z}_{ij}) = \delta _1 I(\textbf{z}_{ij} \in S_1) + \ldots + \delta _t I(\textbf{z}_{ij} \in S_t) \end{aligned}$$

where $\delta _1,\ldots ,\delta _t$ represent the parameter estimates for the single terminal nodes.

**Fig. 1:**
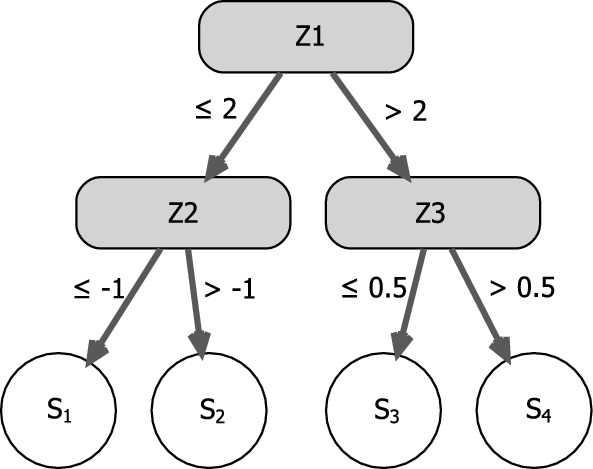
Exemplary illustration of $f(\textbf{z}_{ij})$ as a tree with four terminal nodes $S_1,\ldots ,S_4$

Figure 1 exemplarily shows a representation of $f(\textbf{z}_{ij})$ as a tree with four terminal nodes $S_1,\ldots ,S_4$ where $\textbf{z}$ consists of *p* covariates $Z_1,\ldots ,Z_p$, but only the first three variables $Z_1,\ldots ,Z_3$ are selected for splits. In this example, the tree would be represented as

5 $$\begin{aligned} f(\textbf{z}_{ij}) = \delta _1 I(\textbf{z}_{ij} \in S_1) + \delta _2 I(\textbf{z}_{ij} \in S_2) + \delta _3 I(\textbf{z}_{ij} \in S_3) + \delta _4 I(\textbf{z}_{ij} \in S_4)\,. \end{aligned}$$

Each terminal node can be represented as a product of indicator functions. For example, $S_1$ is denoted as $I(\textbf{z}_{ij} \in S_1)=I(z_{ij1} \le 2)\,I(z_{ij2} \le -1)$.

### Conditional logistic regression forests

In this paragraph, the proposed conditional logistic regression forests (CLogitForest) are introduced. We start by explaining the estimation process before elaborating on their interpretation via variable importance and the potential use of bootstrap confidence intervals for exposure effects.

#### Estimation

CLogitForest is an ensemble learner technique with CLogitTree as base-learner. The estimation is based upon *ntree* bootstrap samples which are sampled from the original training data. A main characteristic of data from matched case–control studies is that they are built up by a number of strata which cannot be separated. Therefore, sampling of the different bootstrap samples has to be done on the level of the *n* strata. Sampling is either performed by regular bootstrap sampling (sampling of *n* strata with replacement) or by taking a subsample of 63.2% of the *n* strata without replacement. The number $63.2\%$ results from the fact that in the case of regular sampling with replacement, the expected number of unique elements is $(1-e^{-1})n\approx 0.632\,n$.

In each of the samples, a separate CLogitTree is estimated. In order to additionally de-correlate the single trees, the parameter *mtry* needs to be specified. In each potential split, only a random subset of *mtry* out of all *p* covariates is used. This guarantees that the different trees will differ even more than already by the fact that they use different samples of the data.

In contrast to single trees, trees serving as base-learners of ensemble methods are allowed to overfit the data. The overfitting of single base-learners (in our case single trees) is compensated by combining them to a joint model (in our case to a random forest). Therefore, within CLogitForest the single trees are not pruned via permutation tests or the Bayesian information criterion (BIC), which were the possibilities of pruning proposed by Schauberger et al. [9]. However, in the accompanying implementation [16] other arguments can be applied to prevent the tree from producing terminal nodes with a too small number of observations (see Sect. 2.4).

A recommended option is to perform the estimation of the conditional logistic regression (underlying each tree from CLogitTree) using an $L_2$ penalty term. Using an $L_2$ penalty has the goal of stabilizing the estimates in cases where perfect separability between cases and controls is achieved within the tree. The penalty term is scaled by a regularization parameter $\lambda$, which is typically set to a small value like $\lambda = 10^{-20}$. For further details on the exact implementation of these arguments see Schauberger et al. [9].

The final forest model is the aggregation of the single trees. Prediction can simply be done by averaging the predictions of the *ntree* different trees. In case a separate exposure effect $\beta$ is estimated, the final estimate for $\beta$ is the average of all single estimates in the trees.

#### Variable importance

The main advantage of using trees instead of forests in general, but also in our particular application to matched case–control studies, is that the resulting models are much more stable than single tree models. Also, the functional relationship between the covariates and the outcome can be much more complicated than in a single tree. The downside of this increased degree of flexibility is, that the interpretation of the functional relationships between covariates and the outcome becomes much harder. Accordingly, random forests are often termed as black-box models which do not allow for any insights of the functional relationships while trees are very intuitive to understand and easy to display.

However, also for black-box models methods of interpretable machine learning [17] exist which can help identify the fitted model’s underlying structures. A popular method to identify the relevance of the various covariates within random forest models is the concept of variable importance [1], which can also be implemented for CLogitForest. In order to measure the variable importance of a particular variable, this variable is permuted within the given training data. Subsequently, the estimated forest (which is based on the non-permuted variables) is used to predict the respective outcome using the permuted variable (while all other variables remain unchanged). In CLogitForest, we use the predictive conditional likelihood on the level of the single strata as the quality measure of the respective prediction. Finally, this predictive conditional likelihood can be compared to the predictive conditional likelihood based on the non-permuted variables. The larger the difference, i.e. the more the predictive accuracy decreases after permutation of a variable, the more important is the variable for the estimated forest model. Two versions of measuring variable importance are implemented in the accompanying R package. The first version is the rather classic version where measuring variable importance is based on all observations for all trees. The second version only relies on out-of-bag observations for each tree. That means, that for each tree only observations are used which are not part of the training data of the respective tree, as they are not part of the respective bootstrap sample. The later version is preferred due to its increased robustness against overfitting to the training data.

If the model incorporates an explicit exposure effect, the variable importance gives us valuable information about which covariates have the highest importance. This can be substantially different to the importance of the single variables we would see in CLR where variables with an important (but non-linear) influence will be neglected. However, this can be highly valuable information for researchers interested in gaining a deeper understanding of the underlying confounding structure.

Furthermore, variable importance measures can also be immensely helpful in cases where an exploratory analysis is applied to a matched case–control study. In such a case, one is usually interested in finding the most important risk factor(s) for the disease at hand from a set of potential candidates. If the association between the risk factors and the outcome is complicated, is potentially non-linear or includes multiple interactions with other variables, this can be detected by CLogitForest much better than by CLR, but also CLogitTree. Additionally, as CLogitForest provides a more stable estimation, it will also lead to a more stable and reliable detection of the most important variables compared to CLogitTree. Therefore, implementing variable importance measures can help to identify important risk (or protective) factors in an exploratory analysis and to distinguish between important and unimportant variables.

#### Confidence intervals

Conventional random forests are not able to provide single parameter estimates and accompanying confidence intervals, as they typically do not contain any global linear parameters. In the method proposed here, the random forest can contain a linear term representing the overall exposure effect. In explanatory analyses of matched case–control studies, this effect is of main interest for researchers. Accordingly, it is important to not only quantify the effect itself but also its uncertainty. We propose to quantify the uncertainty of the exposure effect via nonparametric bootstrap confidence intervals, adapting the concept of confidence intervals for CLogitTree to CLogitForest. The details of the procedure can be found in Schauberger et al. [9]. The main idea is to repeatedly apply the whole procedure of estimating a random forest to a large number of bootstrap samples [4] of the training data. From the resulting estimates of the exposure effect, quantiles can be deduced which represent the corresponding bootstrap confidence interval.

### Implementation

The proposed method is implemented in R within the add-on package CLogitTree [16] and publicly available from https://github.com/Schaubert/CLogitTree.

#### Main functions

The package contains both a function to fit conditional logistic regression trees (CLogitTree()) and a function to fit conditional logistic regression forests (CLogitForest()). Both algorithms can be run parallel on several nodes. The most important parameters to choose in CLogitForest() are ntree (the number of trees) and mtry (the number of randomly selected possible splitting variables). While the number of trees is less important as long as it is chosen large enough, the choice of mtry can have a significant effect on the performance [18]. Therefore, the user is offered an internal tuning procedure for the choice of mtry. Within this procedure, all possible values for mtry between 2 and *p* are cross-validated using a pre-defined number of trees. The predictive out-of-bag conditional likelihood is used as the optimality criterion.

Furthermore, the user can choose specific options for the trees which are fitted within the forest. In particular, there are arguments for the maximal depth of the trees, the minimal node size in order to be eligible for further splitting and the minimum number of observations in any terminal node. For a deeper introduction into the different arguments typically used in random forests and further important aspects for the training of random forests we refer to Boulesteix et al. [19].

#### Supporting functions

The most important supporting functions for CLogitForest() are the function varimp() for calculating and plotting the variable importance as well as the function boot.ci() for calculating bootstrap confidence intervals for the exposure effect.

#### Inclusion of a linear offset

As described above, in case an exposure variable is defined, each tree is initialized with only this exposure effect as a linear effect. However, the initial model could actually be extended. In the software implementation of CLogitForest, an option is offered to include the linear fit of all covariates as an offset before the trees are grown. This offset is the sum of the linear effects of all covariates from CLR but excludes the linear effect of the exposure variable. By using this design, each tree is built upon the fit of CLR before the first split is performed. All potential further splits only have the goal to account for all non-linear effects or interactions which have not yet been captured within the linear fit.

## Results

### Simulation study

A simulation study compares CLogitForest (with and without linear offset) to CLogitTree and CLR. In order to get some insight into the strengths and weaknesses of the methods, we compare different settings for the data-generating process (DGP).

#### Simulation settings

We use three different DGP settings: A, B, and C. Each of the three DGPs is explored together with three combinations of the number of signal and noise variables. The settings we explore are i) 10 signal and 0 noise variables (denoted as 10/0), ii) 5 signal and 5 noise variables (denoted as 5/5), and iii) 10 signal variables and 20 noise variables (denoted as 10/20). For each setting, 100 replications are performed. In all settings, the simulation process follows the same basic structure. They all have in common that we assume a hypothetical population consisting of 500 000 persons, which are randomly distributed to 1000 districts representing the area of residence as a matching variable. Subsequently, the following steps are taken to create a single simulated data set:

$\mathbf {1)}$ Sampling of District Effects:
For each district *j*, we randomly draw two district effects from a uniform distribution between $-2$ and 2. The first district effect $\tau _j$ represents the effect of district *j* on the probability of being diseased $P(y_{ij}=1)$. The second district effect $\tau _j^x$ represents the effect of district *j* on the probability of being exposed $P(x_{ij}=1)$.
$\mathbf {2)}$ Sampling of Person Characteristics:
For each person, depending on the data generating process either 10 or 30 normally distributed person characteristics $\varvec{z}_{ij}$ were sampled, where two of these variables were dichotomized.
$\mathbf {3)}$ Sampling of Exposure Variable:
Based on a linear function of the variables in $\varvec{z}_{ij}$ and the district effects $\tau _j^x$, the probability of being exposed was computed for each person via the logistic function and the exposure variable $x_{ij}$ was sampled (the exact function can be found in the supplement of this manuscript).
$\mathbf {4)}$ Sampling of Outcome Variable:
In order to get the probability of being diseased for each person we computed
  $$\begin{aligned} \eta _{ij} = -2 + \tau _j + \beta \cdot x_{ij} + f_k({\textbf {z}}_{ij}) \end{aligned}$$
  where $\beta = log(2)$ represents the exposure effect. Using the logistic function, $\eta _{ij}$ is transformed into the probability of being diseased, which is used to sample the disease status $y_{ij}$. The function $f_k(\cdot )$ differs between the settings where $k \in \{A, B, C\}$. In setting A, $f_A(\cdot )$ is an additive linear function of the signal variables in $\varvec{z}_{ij}$. In setting B, $f_B(\cdot )$ is the sum of two decision trees, which are based on the signal variables in $\varvec{z}_{ij}$. In setting C, $f_C(\cdot )$ is the sum of smooth functions of the signal variables in $\varvec{z}_{ij}$. In each replication, the order of the signal variables of $\varvec{z}_{ij}$ is permuted so that the true DGP differs in each simulation round. The exact implementations of the functions can be found in the supplement of this manuscript.
$\mathbf {5)}$ Sampling of Final Data Set:
The final data sets consist of 400 strata. First, randomly, 400 of the diseased persons were sampled as cases. Based on the districts of the sampled cases, each case is matched with three controls (randomly selected from the non-diseased persons). Therefore, a data set ultimately consists of 400 strata and a total of 1600 observations.

#### Quality measures

We use two different quality measures for the comparison of the methods. The first quality measure is the predictive conditional likelihood. In order to compute this measure out-of-sample, a new second sample of the respective data set is simulated. For this validation data, the conditional likelihood per stratum is computed and averaged across strata. The higher the predictive likelihood, the better the general fit of the model.

The second quality measure is the absolute errors in the estimation of the exposure effect.

Here, no validation data are needed as the true value of the exposure effect is known within the simulation study.

#### Simulation results

**Fig. 2:**
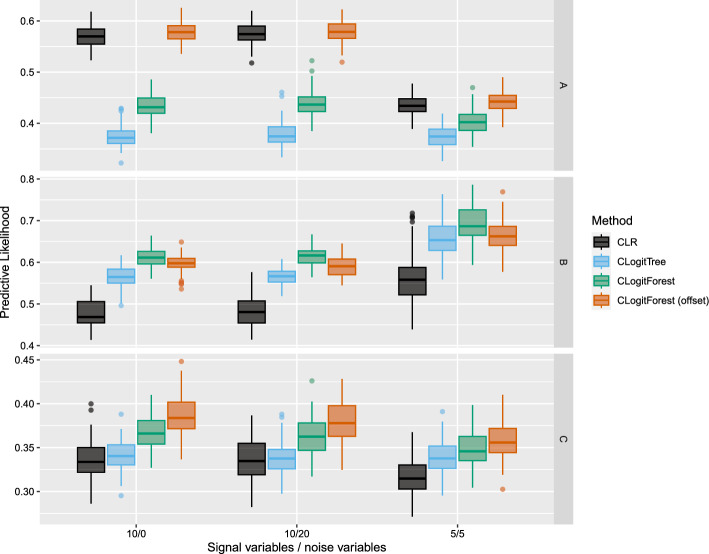
Average predictive conditional likelihood in different simulation settings

Figure 2 displays the average values of the predictive conditional likelihood across the 100 iterations in each simulation setting. We can see that the performance mainly depends on the type of the data generating process and less on the number of signal and noise variables.

Clearly, in the linear setting A, CLogitForest with a linear offset outperforms the version without a linear offset and CLogitTree. CLR performs equally well. In tree setting B, the regular version of CLogitForest performs slightly better than its competitors. All three tree-based methods outperform CLR here. In the smooth setting C, again the tree-based methods perform better than CLR (with CLogitForest with offset as the best method), however, the difference is less clear than in B.

In all settings, both versions of CLogitForest performed better than CLogitTree with respect to the predictive conditional likelihood. This indicates that the forests indeed are better (i.e. more flexibly) able to adapt to different functional relationships, which leads to a better model fit. Accordingly, in an exploratory setting, we could expect the variable importance values from CLogitForest to be more realistic than from CLogitTree. CLR is only a strong competitor with respect to the predictive conditional likelihood if the DGP is linear.

**Fig. 3:**
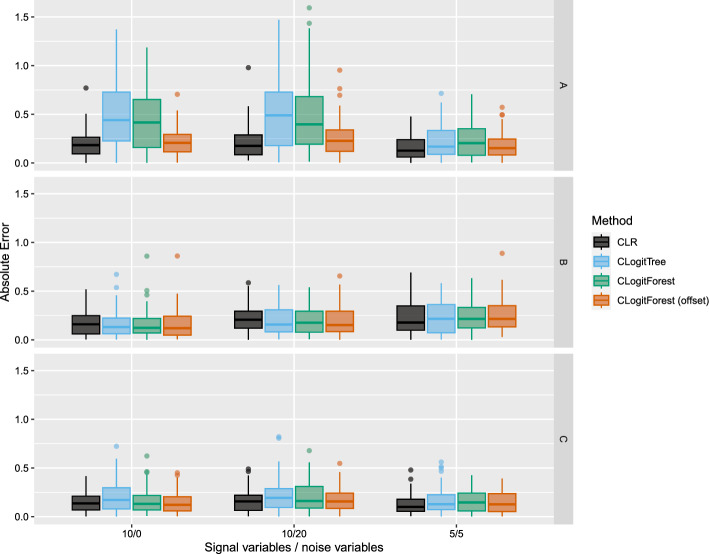
Absolute errors for log exposure effect in different simulation settings

Figure 3 displays the absolute error for estimating the log exposure effect. In the linear setting A, CLogitTree and regular CLogitForest are outperformed by CLR. However, CLogitForest with a linear offset performs equally well as CLR. In settings B and C, we hardly see any major differences. Overall, all methods perform comparably well. The best performance of the tree-based methods can be seen in setting B with 10 signal variables and 20 noise variables, where all three methods perform slightly better than CLR.

### Application to TeQaZ study data

Analogous to the data application in Schauberger et al. [9], the proposed method is illustrated by applying it to data from the TeQaZ study [20]. In this study, women suffering from invasive cervical cancer (ICD-10 C53) were matched to eligible controls based on age (± 2 years) and residence area. The exposure of interest in this study was whether the women frequently participated in cervical cancer screening (*CCS*). The corresponding variable was defined as $CCS=1$ if women had attended *CCS* at least every three years within the past ten years, including at least once in the three years preceding diagnosis, and $CCS=0$ otherwise. In total, the data set comprises 170 cases and 425 controls. Beside the exposure variable *CCS*, several further covariates were considered as potential confounders and predictors which should be included in the data analysis. A listing of all variables from the data set can be found in Table 3 in the supplement of this manuscript.

The goal of this case study is to analyse the TeQaZ data using our newly proposed method CLogitForest and to compare it to CLR and CLogitTree in a real-world data application. However, analogous to the analyses performed in Tanaka et al. [20], for CLR we cannot use the original variables. Many of the variables are categorical (ordinal) variables, which would result in a vast amount of parameters if they were used in their original version in CLR. Therefore, in Tanaka et al. [20] all categorical variables were dichotomized previous to the analysis (compare column *Coding (CLR)* from Table 3 in the supplement of this manuscript). For CLogitTree and CLogitForest, arbitrary dichotomization of variables previous to the actual analysis is not necessary, as these methods inherently find splits between categories during the fitting process. This can be seen as an advantage of the tree-based methods CLogitTree and CLogitForest over CLR. Accordingly, the underlying data in CLR will not be exactly the same as the data used in CLogitForest and CLogitTree.

#### Explanatory analysis

Our first analysis of the data is an explanatory model with a special focus on the dedicated exposure variable *CCS*. CLogitForest is applied both in its default version and with the option of a linear offset. The results are compared to the results of CLogitTree (where BIC is used as pruning criterion), and CLR. For further comparison, a CLR model without covariates is estimated (CLR$^0$). In this and all following analyses, both CLogitForest and CLogitTree will internally be used with a small $L_2$ penalty where $\lambda = 10^{-20}$. Moreover, we set $ntree=500$ and *mtry* is tuned internally as described in Sect. .

**Table 1:** Comparison of parameter estimates and adjusted odds ratios (TeQaZ data)

	CCS effect $\hat{\beta }$	Adj. OR $\exp (\hat{\beta })$	95% CI for $\beta$
CLogitForest	2.106	8.214	1.875–3.224
CLogitForest (linear offset)	1.695	5.447	1.290–2.753
CLogitTree	1.830	6.235	1.355–2.881
CLR	1.586	4.882	0.999–2.172
CLR$^0$	1.806	6.086	1.317–2.295

Estimates for *CCS* (exposure) effect $\beta$ and corresponding adjusted odds ratio with estimates for 95% confidence interval, separately for *CLogitForest*, *CLogitForest* with linear offset, CLogitTree, CLR, and CLR$^0$

Table 1 shows the estimates of the exposure parameter $\beta$ separately for all methods, together with its equivalent as adjusted odds ratio and a corresponding 95% confidence interval. For CLogitForest and CLogitTree, the confidence intervals are estimated via bootstrap using 50 replications.

The results show some diversity with respect to the estimate of the exposure effect of *CCS*, with the largest effects estimated by CLogitForest and the smallest effects estimated by CLR. Of course, the true parameter value is unknown to us. Interestingly, the bootstrap confidence interval from CLogitForest is more narrow compared to the confidence interval from CLogitTree. We suspect that this results from the fact that the estimation process via random forests is generally more stable than via single trees. The results from ClogitForest with a linear offset are closer to the results from CLR, which is also an intuitively plausible results.

**Fig. 4:**
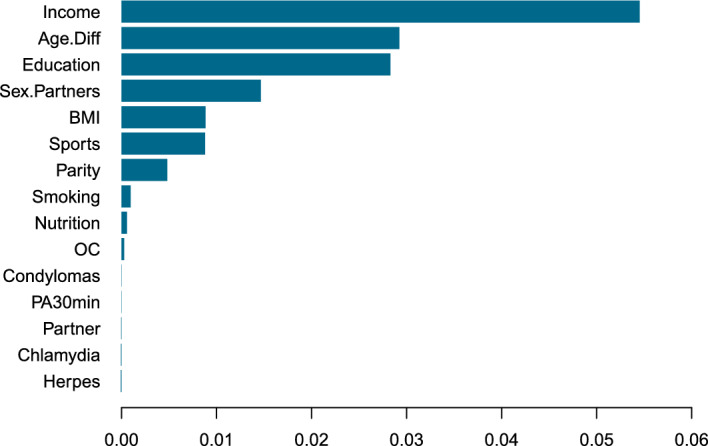
Variable importance for CLogitForest applied to TeQaZ data

Figure 4 displays the (out-of-bag) variable importance for the regular (i.e. not including a linear offset) CLogitForest. The exposure variable *CCS* is missing in this graphic as it is not part of the random forest but modeled separately in a linear effect. The variable *Income* turns out to be the most important of all covariates, followed by Education and Age.Diff. It is well known that a high social status is a preventive factor against cervical cancer [21]. In our analysis, income serves as a proxy for social status.

**Fig. 5:**
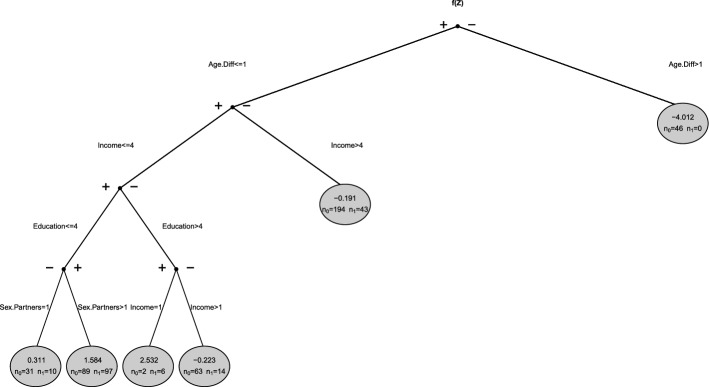
Dendrogram for CLogitTree applied to TeQaZ data. BIC is used as pruning criterion, $L_2$ penalty parameter $\lambda = 1e-20$

For comparison, Fig. 5 shows the dendrogram we get if we analyse the TeQaZ data using CLogitTree.

Interestingly, the first split in the tree is in *Age.Diff*, which is only the third most important variable in CLogitForest. This indicates that random forests may present a more complete picture than a single trees as the first split may not always turn out to be the most important explanatory variables. However, the four variables used for splits by CLogitTree (i.e. *Age.Diff*, *Income*, *Education*, and *Sex.Partners*) also turned out to be the four most important variables in CLogitForest.

#### Exploratory analysis

The second type of analysis represents an exploratory instead of an explanatory analysis.

We claim CLogitForest to be advantageous for exploratory analyses when the functional relationships between potential risk factors and the outcome are unknown, potentially complex, non-linear, or include interactions.

For this exploratory analysis, we for now ignore the fact that this study contains a dedicated exposure variable *CCS*. Rather, we treat *CCS* as all other covariates to be one of several potential risk factors for our disease at hand.

**Fig. 6:**
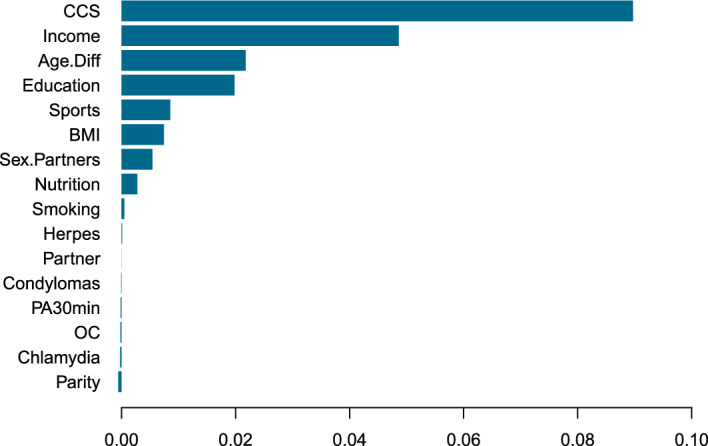
Variable importance for CLogitForest applied to TeQaZ data without specifying *CCS* as the dedicated exposure variable

Figure 6 shows the corresponding variable importance values for an application of CLogitForest to the TeQaZ data without specifying *CCS* as the dedicated exposure variable.

We can see that *CCS* is ranked highest among all variables. Accordingly, in this fictive exploratory data analysis, we would have detected *CCS* to be the by far most influential risk factor among all candidate variables. As *CCS* originally was the exposure variable of interest in this study, this result appears to be particularly reasonable.

#### Model comparison via cross-validation

For comparison, we present a 10-fold cross-validation of this analysis. Using cross-validation, we can see which method performed best with respect to a specific measure, even in a real-world analysis where the true parameter values are unknown. For a general introduction to cross-validation, we refer to Hastie et al. [22]. In our analysis, the data set was randomly split into ten subsets (based on strata level). Iteratively, each subset was once used as the prediction data set while all other subsets were used to build the respective training data. Thus we can compare the observed outcomes to predicted outcomes for all strata while the respective strata have not been part of the respective training data set. The performance measure we used is the (predictive) conditional likelihood per stratum.

**Fig. 7:**
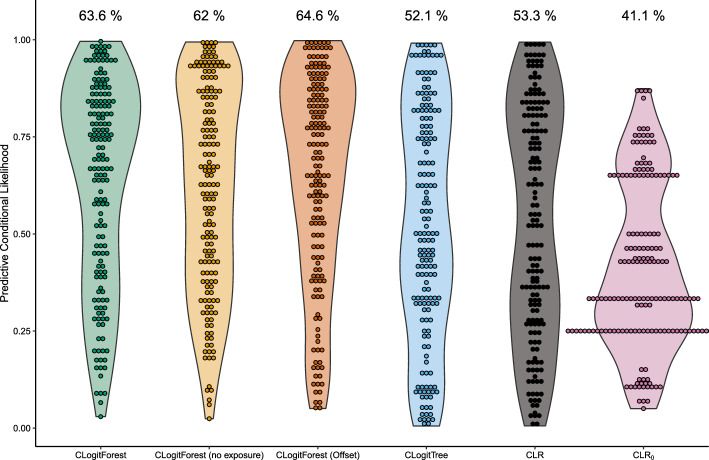
Violin plots for predictive conditional likelihood from 10-fold cross-validation for TeQaZ data, separately for different methods. Numbers on top of violin plots represent average values

Figure 7 shows violin plots of the predictive conditional likelihood results for all strata, separately for the different methods we compare. The average values are 64.3% for CLogitForest, 63.6% for CLogitForest without specifying *CCS* as exposure effect, 65.6% for CLogitForest with linear offset, 52.1% for CLogitTree, 53.3% for CLR and 41.1% for CLR$^0$, where the CLR only contains the exposure variable *CCS*, but no covariates. We can interpret this predictive conditional likelihood as the probability predicted by the model, that (conditional on the fact that each stratum contains exactly one case) the case is the one diseased person from the stratum. The three versions of CLogitForest perform very similar and clearly outperform all other methods. This indicates, that the true data structure is more complicated than captured by a single tree or assuming linearity and that the increased flexibility of forests leads to a drastic improvement in the model fit. Accordingly, choosing one of the three versions of CLogitForest (depending on the exact research question) for the analysis of the TeQaZ data clearly seems to be advantageous both compared to CLogitTree and CLR.

#### Computation time and memory usage

Finally, as an add-on to the comparison using cross-validation presented above, we also present a short comparison in terms of computation time and memory usage between the models estimated via CLogitForest and CLogitTree presented above.

**Table 2:** Comparison of computation time and object size (TeQaZ data)

Method	Computation Time (in seconds)	Size (in MB)
CLogitForest (explanatory)	1412.55	1126.39
CLogitForest (exploratory)	1597.20	1409.00
CLogitTree (BIC)	18.08	0.21
CLogitTree (permutation tests)	59.48	0.60

Computation time (in seconds) and object size (in MB) for two versions of CLogitForest (explanatory and expolartory) and two versions of CLogitTree (pruned using BIC or permutation tests), respectively

Computation was done using parallelization over 20 cores in RStudioServer, running under Ubuntu 22.04.3 LTS. Table 2 contains the computation time in seconds and the size of the resulting objects in R in MB. All of these models have been presented above, except for CLogitTree using permutation tests, which is the alternative to BIC when it comes to pruning in CLogitTree. In this case, the respective tests are based on 500 trees. Clearly, CLogitForest is computationally much more demanding compared to CLogitForest, both with respect to computation time and memory usage.

## Discussion

We present CLogitForest as a machine learning method to analyse matched case–control studies. The method builds upon the concept of CLogitTree as it uses single trees as base learners which build an ensemble learner. CLogitForest shares the advantages of CLogitTree over CLR. These are mainly the increased flexibility with respect to the functional relationship between covariates and outcome as well as the incorporation of interactions between covariates in a data-driven manner. However, a forest overcomes the typical weakness of a single tree of having a comparably high variability. Furthermore, forests are able to learn even more complex functional relationships between the covariates and the outcome than trees. It allows to better adapt to non-linear relationships and to learn arbitrarily complex multivariate surfaces instead of only dealing with multi-dimensional step functions. The increased flexibility comes with the price, that the resulting model is a black-box, which is much harder to interpret. We propose to use variable importance measures in order to allow for some interpretation of the single covariates.

In a simulation study, we showed that CLogitForest performs equally well as CLR when it comes to the estimation of explicit exposure effects within matched case–control studies. However, it clearly outperforms CLR and CLogitTree with respect to the overall model fit. This particularly gives rise to our claim that CLogitForest is a valuable tool for exploratory analyses of matched case–control studies. The real-world application was used to illustrate the use of the proposed method in an exploratory analysis.

Variable importance measures can be used to detect the most important covariates. In the application, it could be seen that CLogitForest in combination with variable importance can enhance our understanding of the importance of the covariates, as the variable with the first split in CLogitTree turned out not to be the most important variable in CLogitForest. In future research, further methods of interpretable machine learning [17] could be exploited in CLogitForest which can help to get a deeper understanding of the functional relationships between the important risk factors and the outcome.

The main intention of this manuscript is to add a further method to the toolbox for the analysis of matched case–control studies. Up to now, CLogitTree was the only machine-learning tool available for this kind of analysis. We see an urgent need for a broader variety of choices for the data analyst. Beside random forests and decision trees, also other machine-learning methods are certainly worth to be explored. A variety of possibilities to analyse matched case–control studies will increase the reliability of the respective results and, therefore, help to gain better evidence from them.

## Conclusion

The study presents CLogitForest as a new machine learning technique for the analysis of matched case–control studies. CLogitForest represents an important contribution as machine learning can open a new range of possibilities for researchers to model matched case–control studies more realistically and, therefore, to gain better insights. Up to now, the range of machine learning techniques for matched case–control studies is very limited, because regular methods fail to take the matching information into account. CLogitForest is based on CLogitTree, which is a tree-based method for modelling matched case–control studies. CLogitForests extends the possibilities provided by CLogitTree as it allows for more stable and more complex functional structures and interactions for the explanatory variables.

## Data Availability

An R implementation for the method introduced in this manuscript is available from Github (https://github.com/Schaubert/CLogitTree). Data sharing is not applicable to this manuscript as no new data were analyzed in this study. Data availability for the TeQaZ data used in this manuscript is described by Tanaka et al. 2021 and can be found under https://doi.org/10.1371/journal.pone.0253801.

## Appendix A functions for outcome sampling in simulation study

In the simulation study in Sect. , different simulation settings were investigated, which differed with respect to the functional relationships between the covariates and the outcome variable. In this appendix, we present the code for the different functions $f_k(z_{ij})$ in the simulation settings A, B, and C. Each function takes the following arguments:

Z Matrix of dimension $N\times p$ (where $N=\sum _{i=1}^n m_i$ and $p = p.signal + p.noise$) containing all covariates (not the exposure) for all observations
p.signal Number of signal variables
p.noise Number of noise variables

In each of the settings A, B, and C, the functions distinguish between the settings

i) p.signal = 10, p.noise = 0
ii) p.signal = 5, p.noise = 5
iii) p.signal = 10, p.noise = 20

### Setting A

Setting A represents a setting where $f_A(z_{ij})$ consists of additive linear functions of the single variables. The R-code of the corresponding function is found below:

### Setting B

Setting B represents a setting where $f_B(z_{ij})$ consists of two additive trees. The R-code of the corresponding function is found below:

### Setting C

Setting C represents a setting where $f_C(z_{ij})$ consists of additive smooth functions of the single variables. The R-code of the corresponding function is found below:

## Appendix B Variable description of TeQaZ data

See Table 3.

**Table 3:** Variable description of TeQaZ data

Variable	Description	Coding	Coding (CLR)
ID	Case-control strata ID	$1,\ldots ,170$	$1,\ldots ,170$
CCS	Frequent participation in CCS	1: yes, 0: no	1: yes, 0: no
Smoking	Ever smoked	1: yes, 0: no	1: yes, 0: no
Education	Highest school degree	1: no degree 2: Hauptschule 3: Realschule 4: Polytechn. Oberschule 5: Fachoberschule 6: Gymnasium/Abitur	1: Fachoberschule / Gymnasium 0: else
Income	Net monthly household income	1: $<1250$€ 2: 1250€–1749€ 3: 1750€–2249€ 4: 2250€–2999€ 5: 3000€–3999€ 6: 4000€–4999€ 7: $\ge 5000$€	1: $\ge 3000$€ 0: $< 3000$€
BMI	Body mass index	in $kg/m^2$	in $kg/m^2$
Partner	Currently living with partner	1: yes, 0: no	1: yes, 0: no
OC	Ever used oral contraceptives	1: yes, 0: no	1: yes, 0: no
Sports	Sporting activity	0: Never 1: $\le$ 1–3 times/month 2: 1–2 times/week 3: 3–4 times/week 4: $\ge$5 times/week	1: $\ge$ 1 time/week 0: < 1 time/week
Parity	Number of children		1: $\ge 4$ children 0: $<4$ children
Sex.Partners	Number of sexual partners	1: 1 partner 2: 2–3 partners 3: 4–10 partners 4: $\ge 10$ partners	1: $> 1$ partners 0: 1 partner
Nutrition	Portions of fruit and vegetables/day	0: none 1: 1–2 portions 2: 3–4 portions 3: $\ge$5 portions	1: $\ge 3$ portions 0: $<3$ portions
Herpes	Ever genital Herpes infection	1: yes, 0: no	1: yes, 0: no
Chlamydia	Ever Chlamydia infection	1: yes, 0: no	1: yes, 0: no
Condyloma	Ever Condyloma infection	1: yes, 0: no	1: yes, 0: no
PA30min	30 min physical activity/day	1: yes, 0: no	1: yes, 0: no
Age.Diff	Age–average age in stratum	in years	in years
Y	Case-Control status	1: Case, 0: Control	1: Case, 0: Control

‘Coding’ refers to coding for *CLogitForest* and *CLogitTree*, ‘Coding (CLR)’ refers to coding for CLR

## Abbreviations

BIC: Bayesian information criterion
CART: Classification and regression trees
CLogitForest: Conditional logistic regression forest
CLogitTree: Conditional logistic regression tree
CLR: Conditional logistic regression
DGP: Data-generating process
RWD: Real-world data
RWE: Real-world evidence
